# Chronic Kidney Disease Management in General Practice: A Focus on Inappropriate Drugs Prescriptions

**DOI:** 10.3390/jcm9051346

**Published:** 2020-05-04

**Authors:** Maria Antonietta Barbieri, Michelangelo Rottura, Giuseppe Cicala, Rossella Mandraffino, Sebastiano Marino, Natasha Irrera, Carmen Mannucci, Domenico Santoro, Francesco Squadrito, Vincenzo Arcoraci

**Affiliations:** 1 Department of Clinical and Experimental Medicine, University of Messina, 98125 Messina, Italy; mbarbieri@unime.it (M.A.B.); mrottura@unime.it (M.R.); gcicala@unime.it (G.C.); nirrera@unime.it (N.I.); dsantoro@unime.it (D.S.); fsquadrito@unime.it (F.S.); 2 General Practitioner, Provincial Health Unit of Messina, 98100 Messina, Italy; rossellamd@gmail.com (R.M.); steniomarino@gmail.com (S.M.); 3 Department of Biomedical and Dental Sciences and Morpho-functional Imaging, University of Messina, 98125 Messina, Italy; cmannucci@unime.it

**Keywords:** chronic kidney disease, appropriateness of prescriptions, prescribing patterns, real-world data, general practice

## Abstract

Nephrotoxic drugs prescriptions are often prescribed inappropriately by general practitioners (GPs), increasing the risk of chronic kidney disease (CKD). The aim of this study was to detect inappropriate prescriptions in patients with CKD and to identify their predictive factors. A retrospective study on patients with creatinine values recorded in the period 2014–2016 followed by 10 GPs was performed. The estimated glomerular filtration rate (eGFR) was used to identify CKD patients. The demographic and clinical characteristics and drugs prescriptions were collected. A descriptive analysis was conducted to compare the characteristics and logistic regression models to estimate the predictive factors of inappropriate prescriptions. Of 4098 patients with creatinine values recorded, 21.9% had an eGFR <60 mL/min/1.73 m^2^. Further, 56.8% received inappropriate prescriptions, with a significantly lower probability in subjects with at least a nephrologist visit (Adj OR 0.54 (95% CI 0.36–0.81)) and a greater probability in patients treated with more active substances (1.10 (1.08–1.12)), affected by more comorbidities (1.14 (1.06–1.230)), or with serious CKD (G4/G5 21.28 (7.36–61.57)). Nonsteroidal anti-inflammatory drugs (NSAIDs) were the most used contraindicated drugs (48.5%), while acetylsalicylic acid was the most inappropriately prescribed (39.5%). Our results highlight the inappropriate prescriptions for CKD authorized by GPs and underline the need of strategies to improve prescribing patterns.

## 1. Introduction

Chronic kidney disease (CKD) is one of the most widespread diseases and it is deemed to be a real public health problem worldwide [1]. CKD is defined as deficits in the kidney structure or function that persist for at least three months with consequences for the sufferer’s health. CKD is classified into stages based on glomerular filtration rate (GFR) values and albuminuria measurements, including the albumin excretion rate (AER) and the albumin-to-creatinine ratio (ACR). The normal GFR in adults is about 125 mL/min/1.73 m^2^. GFR values <60 mL/min/1.73 m^2^ indicate a CKD classified as G3a, while values of GFR <15 mL/min/1.73 m^2^ define a kidney failure (category G5) [2].

The Global Burden of Disease study data showed that the incidence and the prevalence of CKD globally increased by 89% and by 87% from 1900 to 2016, respectively [3]. In more detail, CKD has an estimated global prevalence of between 11% and 13%, mainly related to stages G3a and G3b [4]. Nevertheless, a substantial difference between each single country has been observed with a country median prevalence of 8.9% [5,6]. The Italian prevalence of CKD is accounted for in 8.1% of men and 7.8% of women, despite advanced age and an unfavorable cardiovascular risk [7,8]. Moreover, the percentage change in age-standardized rates between 1990 and 2017 was −9.2% in Italy compared with −2.7% registered in Central Europe [9].

Epidemiological data available in the last decades has sparked interest in CKD from health authorities, nephrologists, and general practitioners (GPs). GPs are certainly the first category of physicians able to identify CKD early in patients, taking into account risk factors that could induce a renal failure. They can also take measures timely to slow the progression of the disease and promptly send the patient to a nephrologist [10,11]. The indications for a nephrology referral are different among countries, although these indications always recommend to refer all patients with an eGFR <30 mL/min/1.73 m^2^ [2]. In Italy, the National Health System suggests that patients could be referred to a nephrologist also when their eGFR is less than 60 mL/min/1.73 m^2^ and with at least one of the following conditions: progressive worsening of renal function (eGFR <15% in 3 months), diabetes mellitus, or aged <70 years [12]. GPs’ delays in referring patients to the nephrologist might cause the worsening of their renal function [13]. Unfortunately, GPs’ awareness of CKD is very poor in Italy and a strong cooperation between primary care physicians and nephrologists is needed to implement early strategies and planning interventions in CKD [14].

Furthermore, the presence of CKD could be associated with several chronic diseases including hypertension, diabetes, chronic respiratory disorder, cardiovascular diseases, and depression [15,16,17]. Patients with at least one comorbidity listed above are usually in treatment with several drugs that are mostly metabolized and eliminated by the kidney. The altered pharmacokinetics of drugs renally excreted as well as an increased risk of drug interactions are frequent in patients with CKD and their dosages require adjustment in order to avoid toxicity [2]. Therefore, polytherapy could lead to greater difficulties in pharmacological management and the inappropriate prescribing of contraindicated medications could result in the worsening and progression of CKD [18]. Renin-angiotensin-system (RAS) blocking agents, beta-blockers, antibiotics, lithium, analgesics, nonsteroidal anti-inflammatory drugs (NSAIDs), anticoagulants, hypoglycemic, and some chemotherapeutic agents are known to be nephrotoxic drugs [19,20,21]. Moreover, NSAIDs result as the most commonly prescribed nephrotoxic drugs in CKD patients [22].

Guidelines strongly recommend to avoid the prescription of drug-induced nephrotoxicity principally in patients with several conditions [2]. However, an Italian retrospective population-based study showed that 49.8% and 45.2% of patients received at least one prescription of nephrotoxic drugs within one year before and after the first CKD diagnosis, respectively [23]. The management of CKD by GPs cannot be underestimated, especially because more therapeutic options have been made available in recent decades [24]. Greater familiarity with nephrotoxic drugs by GPs could help to identify appropriate preventive strategies for the management of patients affected by CKD. For the reasons described above, the aim of this study was to detect the prescriptions of nephrotoxic and contraindicated drugs in patients with CKD and to identify their predictive factors in a real-world context.

## 2. Materials and Methods

### 2.1. Study Design and Data Collection

A retrospective observational study was carried out by the Department of Clinical and Experimental Medicine of the University of Messina with a group of 10 GPs of Messina. All patients of each GP aged ≥18 years in the three-year period from 2014–2016 were recruited. Of them, subjects with registered serum creatinine levels in the GPs’ medical records during the considered period were selected.

In detail, the following data concerning patients’ clinical and demographic characteristics were collected:

- Encrypted patient code, age, sex, weight, height, and body mass index (BMI);
- Information on lifestyles, such as alcohol use and smoking;
- Diagnostic instrumental and laboratory exams, of which serum creatinine, blood count, plasma glucose (FPG), glycated hemoglobin (HbA1c), lipid profile, hepatic cytolysis indexes, electrolytes, and albuminuria were examined;
- All drugs prescriptions registered in the GPs’ medical records and classified according to the Anatomical Therapeutic and Chemical (ATC) Classification System;
- Registered diagnosis of CKD and comorbidities codified using the International Classification of Diseases code, 9th revision (ICD-9);
- Requests for specialist visits.

The Charlson comorbidity index score (CH index) was adopted to evaluate the severity of the disease for each patient. The total number of drugs prescriptions as well as the number of different active substances used for any disease by patients during the study period were considered to assess the drugs exposure.

Regardless of the GPs’ recorded diagnosis of CKD, patients were classified according to the Kidney Disease Outcomes Quality Initiative (KDOQI) guidelines based on GFR values estimated with a CKD-epidemiology collaboration (CKD-EPI) formula (eGFR). Patients with an eGFR <60 mL/min/1.73 m^2^ calculated on consecutive registered serum creatinine values at least after 90 days were identified as CKD-affected patients and classified into stages G3a–G5 according to the last recorded eGFR value. In particular, stage G3a (eGFR 45–59 mL/min/1.73 m^2^), G3b (eGFR 30–44 mL/min/1.73 m^2^), G4 (15–29 mL/min/1.73 m^2^), and G5 (eGFR <15 mL/min/1.73 m^2^) were considered [2]. Clinical and demographic characteristics of the CKD-affected patients (G3a–G5) were compared with patients with an eGFR ≥60 mL/min/17.3 m^2^.

A patient encrypted code has been used to maintain anonymity. The study protocol was approved by the local Ethical Committee of Messina University Hospital.

### 2.2. Definition of Nephrotoxicity

For the identification of nephrotoxic drugs, literature reviews were performed using the Medical Subject Headings (MeSH) terms “nephrotoxic drug” and “drug-induced renal failure”. Moreover, an evaluation of the cautionary notes for prescriptions in people with CKD was executed [2]. Specifically, all drugs were classified as “contraindicated drugs” in CKD when taking into account their Summary of Product Characteristics (SmPC) available at the time of the study [25] (see Supplementary Table S1).

### 2.3. Statistical Analyses

A descriptive analysis was performed to compare all characteristics of the study population (e.g., age, sex, comorbidity, nephrologist visits, number of prescriptions) between patients with an eGFR <60 mL/min/1.73 m^2^ vs. patients with an eGFR ≥60 mL/min/1.73 m^2^.

Absolute and relative frequencies with corresponding 95% confidence intervals (CIs) were evaluated for the categorical variables, while medians with an interquartile range (Q1–Q3) were calculated for continuous variables. The Kolmogorov–Smirnov test for normality was performed to evaluate the normal distribution. Since some of the numerical variables were not normally distributed, a non-parametric approach was used. The Mann–Whitney U test for independent samples was applied for the continuous variables and a two-tailed Pearson chi-squared test for the categorical variables.

To identify the predictors of inappropriate prescriptions in patients affected by a stage of CKD ≥G3a, a univariate logistic regression model, using patients without inappropriate prescriptions as comparators, was carried out to assess the possible influence of sex, age, number of nephrologist visits, CKD diagnosis, CH index, stage of CKD, number of comorbidities, number of active substances used, and number of total prescriptions for each patient. Moreover, all predictors were included in a stepwise multivariate logistic regression model (backward procedure, α = 5%). Odds ratios (ORs) with 95% CIs were calculated for each covariate of interest in the univariate (crude OR) and multivariate (adjusted OR) models. The goodness of fit of the regression model was assessed by the Hosmer–Lemeshow test for adequacy. A *p*-value < 0.05 was considered statistically significant. The statistical analysis was performed with SPSS version 23.0 (IBM Corp., SPSS Statistics, Armonk, NY, USA).

## 3. Results

### 3.1. Characteristics of Patients

A total of 13,971 patients were followed by 10 GPs of which 4098 (29.3%) had at least a registered serum creatinine value in the last three years with a high variability among each single GP (from 12.3% to 48.5%). eGFR values were calculated by using the CKD-EPI formula: 3202 subjects (78.1%) had an eGFR of 60 mL/min or more. Patients with an eGFR <60 mL/min/1.73 m^2^, defined as CKD patients, were 896 in number (21.9%). However, only in 29.6% of them did GPs record the diagnosis of CKD during the study period, and only 25.5% of CKD patients were referred to a nephrologist. The frequency of nephrologist visits as well as GPs’ diagnosis of CKD (G3a–G5) increased at the increasing CKD stage (see Supplementary Table S2).

The comparative analysis of the characteristics between the patients with or without CKD according to the eGFR values showed that patients with an eGFR <60 mL/min/1.73 m^2^ were mainly females, older, and affected by more serious diseases, evaluated by the CH index (*p* < 0.001). Furthermore, subjects with an eGFR <60 mL/min/1.73 m^2^ had a greater number of total drugs prescriptions, as well as more different active substances prescribed per patient compared with subjects with an eGFR ≥60 mL/min/1.73 m^2^ (*p* < 0.001) (Table 1). All comorbidities were more frequent in patients with an eGFR <60 mL/min/1.73 m^2^ (*p* < 0.001 for all comparisons). Moreover, patients with an eGFR <60 mL/min/1.73 m^2^ were mostly affected by hypertension (87.6%), dyslipidemia (60.0%), arthritis and arthrosis (56.1%), chronic pulmonary disease (47.2%), and osteoporosis (45.6%) (Table 1).

An increased number of prescriptions was found with an increased stage of CKD (G3a–G5): G3a: 113 (60–187); G3b: 152 (91–229); G4/G5: 196 (117–65), as well as the number of different active substances prescribed: G3a: 18 (12–25); G3b: 22 (15–29); G4/G5: 25 (20–33). The frequency of comorbidities stratified by the severity of CKD are shown in Supplementary Table S2.

### 3.2. Characteristics of Drug Prescriptions

Of 4098 patients with registered serum creatinine values in the last three years, 4018 (98.1%) had at least one drug prescription. Among them, the drugs for acid related disorders, agents acting on RAS, lipid modifying agents, antithrombotic agents, and drugs used in diabetes were the five most frequently prescribed classes.

**Figure 1:**
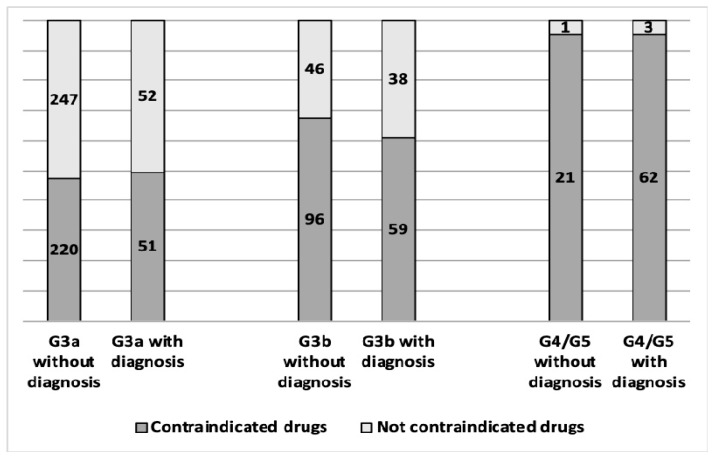
The graph represents patients with or without a diagnosis of chronic kidney disease (CKD) with at least one prescribed contraindicated drug for each stage codified by an eGFR category: G3a between 45 and 59 mL/min/1.73 m^2^; G3b between 30 and 44 mL/min/1.73 m^2^; and G4/G5 < 30 mL/min/1.73 m^2^.

All 896 patients with an eGFR <60 mL/min/1.73 m^2^ received at least one drug prescription with a total of 128,996 prescriptions during the three-year study period. More than half of the patients with an eGFR <60 mL/min/1.73 m^2^ (*n* = 509; 56.8%) received at least one prescription of contraindicated drugs for a total of 4818 (3.7%) inappropriate prescriptions. The median (Q1–Q3) number of contraindicated prescribed medications per patient was 5 (2–14). The number of patients with inappropriate prescriptions increased with the increased CKD G3a–G5 stages, specifically, 47.5% in stage G3a, 64.9% in G3b, and 95.4% in G4/G5. Recording the diagnosis of CKD did not significantly affect the percentage of patients with inadequate prescriptions in every stage of CKD (Figure 1).

The probability of receiving contraindicated drug prescriptions in patients with an eGFR <60 mL/min/1.73 m^2^ was significantly lower in subjects with at least a nephrologist visit (Adj OR 0.54 (95% CI 0.36–0.81), *p* = 0.003). Furthermore, a greater probability of receiving an inappropriate prescription was observed in patients treated with more different drugs (Adj OR 1.10 (95% CI 1.08–1.12), *p* < 0.001) or affected by a higher number of comorbidities (Adj OR 1.14 (95% CI 1.06–1.23), *p* = 0.001). The severity of CKD was also an independent predictive factor of inappropriate prescriptions (Adj OR G3b 1.84 (95% CI 1.29–2.62), *p* = 0.001; Adj OR G4/G5 21.28 (95% CI 7.36–61.57), *p* < 0.001). Nevertheless, sex, age, CH index, the number of prescriptions, and the presence of a registered diagnosis of CKD were not predictive factors of prescriptive inappropriateness (Table 2).

The most commonly used contraindicated drug classes in patients with an eGFR <60 mL/min/1.73 m^2^ were anti-inflammatory and antirheumatic products, and antithrombotics were used at least once by 435 (48.5%) and 161 (18.4%) patients, respectively. Moreover, 2026 (52.8%) of all prescriptions related to anti-inflammatory drugs and 1917 (19.1%) referring to antithrombotic drugs were contraindicated (Table 3).

Regarding single active substances, overall, 31.1% and 19.4% of patients with an eGFR <60 mL/min/1.73 m^2^ took diclofenac and acetylsalicylic acid, respectively. However, the most contraindicated medication prescribed in the total of the inappropriate prescriptions was acetylsalicylic acid (39.5%) followed by diclofenac (17.7%). The contraindicated drugs most commonly prescribed per patient and the related contraindicated prescriptions are shown in Table 4.

## 4. Discussion

This is the first study concerning the evaluation of the predictive factors of contraindicated drug prescriptions in patients with CKD in a general practice setting. To the best of our knowledge, a previous study specifically identified the predictors of NSAIDs use and only recently did a few studies focusing on inappropriate drug prescriptions in patients followed by nephrologists become available [20,23,24,26,27].

Our results confirmed the poor attention in monitoring renal function and inadequate awareness of CKD management by GPs. However, these results bode better than those observed in other studies, which showed at least one registration of a serum creatinine dosage in only 17% of patients compared with 29.3% in our sample, as well as a correct diagnosis of CKD in only 15.2% and 23.2% of patients with an eGFR <60 mL/min/1.73 m^2^ instead of the 29.6% in our sample. Regarding the registered diagnosis of CKD in patients with an eGFR <60 mL/min/1.73 m^2^, our analysis showed a lower focus of CKD, especially in subjects in stage G3a, with a percentage that raised from 18.1% to 74.7% with the increasing severity of CKD. A similar trend has also been observed with regard to nephrological visits in agreement with previous studies [14,28].

In our setting, patients with an eGFR <60 mL/min/1.73 m^2^ were mainly females, older, with more comorbidities, in particular affected by hypertension, dyslipidemia, arthritis and arthrosis, chronic pulmonary disease, and osteoporosis. In addition, an increased age and frequency of chronic diseases such as cerebrovascular disease, diabetes mellitus, heart failure, gout, and metabolism disorders have been found with the increased severity of CKD stages (see Supplementary Materials Table S2). In the literature, it is well known that women are more affected by CKD. It could be explained by the differences in kidney pathophysiology but also by a possible over-diagnosis of CKD due to the incorrect use of the CKD-EPI formula [29,30]. The elderly have a higher prevalence of risk factors related to the onset and the progression of CKD [31]. Furthermore, older people daily take a broad number of drugs, thus increasing the probability of inappropriate prescriptions [32,33]. Multimorbidity was very common in patients with CKD and the number of comorbidities was linked with a faster decline of the renal function [34]. Hypertension and dyslipidemia were the most observed diseases in other studies [17,34]. Furthermore, CKD was one of the most frequent comorbidities in chronic pulmonary disease patients with a significant impact on the outcomes and mortality [35]. Secondary hyperparathyroidism due to the loss of renal function in patients with CKD causes alterations in bone metabolism, developing in an increased risk of osteoporosis and it has led to the definition of the “mineral and bone disorder related to chronic kidney disease” (CKD-MBD) [36]. Our findings showed a higher number of prescriptions and more different active substances taken in patients with an eGFR <60 mL/min/1.73 m^2^ compared with patients with an eGFR ≥60 mL/min/1.73 m^2^. This could be explained by the comorbidities data described above. Indeed, the presence of more chronic diseases could lead to polytherapy with a great number of drugs used for each patient [18]. More than half of our subjects received at least one contraindicated prescription as observed in a previous study [26]. In addition, the proportion of contraindicated prescriptions was greater in patients in the G4/G5 CKD stages compared with stages G3a and G3b, as shown in another study [26]. However, a recent study found that G2–G3 patients received significantly more contraindicated drugs prescriptions than late-stage CKD subjects [20].

As previously observed, the diagnosis of CKD by GPs did not reduce the probability of receiving at least one prescription of nephrotoxic drugs along with the new diagnosis [23]. Once again, this finding confirms the inadequate awareness of GPs on the potential damaging effects of inappropriate drugs prescriptions in CKD patients, that could seriously endanger life. Indeed, GPs maintained the same prescriptive behavior, as also assessed with the multivariate logistic regression analyses. On the contrary, at least one nephrologist specialist counselling was related to a lower risk of receiving contraindicated prescriptions. This result was in accordance with that observed in a study focusing on the use of nephrotoxic medications in older people [27], although the number of counselling per patient did not influence the probability of the inappropriateness, as well as the number of GP visits [26]. Our findings suggest that nephrologist specialist counselling should be considered by GPs from the earliest stage of CKD, as it improves disease management and also reduces the risk of prescribing inappropriate drugs. The number of different active substances taken, the number of comorbidities, and the severity of CKD, unlike the number of total prescriptions, were related to a higher risk of inappropriateness. Further evidence showed that the number of several medications was the most predictive factor of an inappropriate prescription [26,27]. Contrary to our data, comorbidities did not affect the likelihood of inappropriate prescriptions in hospitalized elderly patients [20]. However, the different context has to be taken into account.

The use of inappropriate prescriptions related to the severity of CKD was contradictory. In line with other findings in general practices [26], in our study, the CKD stage was an independent predictor of contraindicate prescriptions, as highlighted in the logistic regression analysis. Alternatively, in the context of hospitalized patients, an inverse association was found [20]. In contrast to the evidence, age was not a predictive factor of inappropriateness. In two previous studies, old age people were less likely to have inappropriate prescriptions, maybe for more accurate attention by GPs on kidney function [26,27]. Nevertheless, another study highlighted a higher probability of inappropriate prescriptions in the elderly [20]. The relation between inappropriate prescriptions in CKD patients and gender was inconsistent. In accordance with other findings, no association between gender and contraindicated prescriptions was noticed [20]. However, a relationship between females or males and inappropriate prescriptions has also been reported in different studies [26,27].

Anti-inflammatory and antirheumatic products and antithrombotics were the most widely inappropriately prescribed drugs in our study. In more detail, one-third of patients with CKD had at least one contraindicated prescription of diclofenac; additionally, 19.4% and 14.0% of subjects received at least one contraindicated prescription of acetylsalicylic acid and etoricoxib, respectively. Furthermore, acetylsalicylic acid was the most inappropriately prescribed medication of all contraindicated prescriptions followed by diclofenac, etoricoxib, and metformin. A higher use of acetylsalicylic acid, diclofenac, and coxib in patients with CKD was observed in other studies [20,23,37]. It is well known that different drugs, including NSAIDs, have a crucial role in the occurrence of nephrotoxic effects induced by the decrease in GFR values and vasodilatation. These effects develop through the inhibition of renal prostaglandin or through other mechanisms including the development of membranous glomerulonephropathy, type 4 renal tubular acidosis, or acute and chronic renal papillary necrosis [38]. CKD-affected patients treated with low-dose acetylsalicylic acid because of cardiovascular prevention had an abnormal platelet function that could increase the risk of hemorrhage. Low-dose acetylsalicylic acid was commonly prescribed inappropriately in patients with CKD. However, CKD increased the risk of cardiovascular disease and mortality. For these reasons, the risk/benefit profile of aspirin is acceptable in this population [23]. Additionally, the use of metformin must always be avoided in patients with severe CKD, especially for an increased risk of lactic acidosis [39]. Nevertheless, in general practice, a high percentage of antidiabetic drugs prescribed in the late stages of CKD that regarded metformin [40] showed that it was one of the three drugs most inappropriately prescribed in patients with an eGFR <30 mL/min/1.73 m^2^ [27].

### Strengths and Limitations

The present study has several main strengths and limitations. The major strength is that we conducted the first overview of the predictive factors of contraindicated drugs prescriptions in a large cohort of patients affected by CKD, in a general practice setting, and in a long-time study period. Alternatively, other studies focused on nephrology departments or on a single drug class [20,23,26]. However, we could not ignore that these results are not entirely generalizable to the Italian general population. We took into account eGFR values calculated by the CKD-EPI formula to detect CKD as it was conducted in other studies [26,27]. However, we considered only patients starting from the G3a stage and all patients in the G2 stage were not taken into account. Moreover, the lack of completed data, particularly regarding the albuminuria laboratory values, did not allow us to select all patients with CKD. A diagnosis registered by GPs might not be accurate even if we used specific ICD-9 codes for CKD. Moreover, all the information was collected from GPs’ medical records and all data came from the clinical practice. As a consequence, several assays from different laboratories could have been used. Nevertheless, the exams considered in this survey are not affected by a wide variability. Some nephrotoxic drugs do not require a GP prescription for their administration in the hospital setting or for their use as over-the-counter (OTC) drugs; for these reasons, the number of contraindicated prescriptions in CKD could be underestimated. No information concerning the source of prescription is registered in the medical records. For this reason, it is not possible to known if prescriptions were directly carried out by the GP or suggested by the specialist. However, in Italy, all treatments are registered in medical records, with most of them also being suggested by specialists, are prescribed by GPs to be free from charge of the citizen, and GPs can obviously decide to prescribe the suggested drug or not.

## 5. Conclusions

Our findings suggest that CKD is not optimally framed in general practices. GPs have a crucial role regarding prescriptions for people with CKD. Nevertheless, the number of contraindicated prescriptions is very high in these subjects, despite the awareness of the disease by GPs. The use of nephrotoxic drugs in patients with CKD may result in increased renal damage with the worsening and progression of CKD that could lead to serious consequences for the sufferer’s health. For these reasons, the dissemination of information and guidelines as well as specific training courses aimed to properly diagnose CKD should be implemented. Moreover, more efficient drug information is needed in order to avoid contraindicated prescriptions in patients with CKD, especially when therapeutic alternatives are available. Lastly, a closer collaboration with nephrologists, not only in the late stages of CKD, might be effective in implementing preventive measures aimed to slow the disease progression and to improve drug management in CKD.

## Figures and Tables

**Table 1:** Characteristics of patients with an estimated glomerular filtration rate (eGFR) ≥60 mL/min/1.73 m^2^ vs. patients with an eGFR <60 mL/min/1.73 m^2^.

Characteristic	eGFR ≥ 60 mL/min/1.73 m^2^	eGFR < 60 mL/min/1.73 m^2^	*p*-Value
**Sex, *n* (%)**			
Male	1695 (52.9)	315 (35.2)	<0.001
Female	1507 (47.1)	581 (64.8)	
Median age (Q1–Q3)	59 (46-69)	77 (70–83)	<0.001
**Age categories, *n* (%)**			
18–65	2106 (65.8)	142 (15.8)	
65–80	923 (28.8)	448 (50.0)	
>80	173 (5.4)	325 (36.3)	
**Comorbidities, *n* (%)**			
Hypertension	1726 (53.9)	785 (87.6)	<0.001
Dyslipidemia	1386 (43.3)	538 (60.0)	<0.001
Arthritis and arthrosis	1263 (39.4)	503 (56.1)	<0.001
Chronic pulmonary diseases	1298 (40.5)	423 (47.2)	<0.001
Osteoporosis	791 (24.7)	409 (45.6)	<0.001
Psychosis	1157 (36.1)	389 (43.4)	<0.001
Diabetes Mellitus	669 (20.9)	322 (35.9)	<0.001
Cerebrovascular disease	534 (16.7)	305 (34.0)	<0.001
Atherosclerosis	254 (7.9)	188 (21.0)	<0.001
Gout and metabolism disorders	182 (5.7)	134 (15.0)	<0.001
Heart failure	79 (2.5)	113 (12.6)	<0.001
Malignant neoplasm	274 (8.6)	110 (12.3)	<0.001
Registered CKD diagnosis, *n* (%)	111 (3.5)	265 (29.6)	<0.001
Nephrologist visits ^1^, *n* (%)	90 (2.8)	228 (25.4)	<0.001
CH index, median (Q1–Q3)	2 (0–3)	3 (1.8–6)	<0.001
Number of prescriptions, median (Q1–Q3)	44 (13–99)	127 (72–205)	<0.001
Number of active substances, median (Q1–Q3)	11 (6–18)	20 (13–27)	<0.001

Abbreviations: CH = Charlson, CKD = chronic kidney disease, eGFR = estimated glomerular filtration rate, Q1 = first quartile, and Q3 = third quartile. ^1^ Patients with almost one nephrologist visit during the study period.

**Table 2:** Predictive factors associated with contraindicated prescription in patients with an eGFR <60 mL/min/1.73 m^2^.

Variables	Crude OR (95% CI)	*p*-Value	Adjusted OR ^1^ (95% CI)	*p*-Value
Sex (F)	1.22 (0.93–1.61)	0.160	1.05 (0.75–1.46)	0.794
Age (years)	1.025 (1.01–1.04)	<0.001	0.99 (0.98–1.01)	0.227
Nephrologist visits	1.66 (1.21–2.26)	0.002	0.54 (0.36–0.81)	0.003
Registered CKD diagnosis	1.61 (1.20–2.17)	0.002	0.83 (0.52–1.34)	0.449
CH index	1.10 (1.06–1.15)	<0.001	0.97 (0.91–1.02)	0.223
CKD stage G3a ^2^	1		1	
CKD stage G3b ^2^	2.04 (1.49–2.78)	<0.001	1.84 (1.29–2.62)	0.001
CKD stage G4/G5 ^2^	22.89 (8.28–63.30)	<0.001	21.28 (7.36–61.57)	<0.001
Number of diseases	1.31 (1.23–1.40)	<0.001	1.14 (1.06–1.23)	0.001
Number of drugs	1.12 (1.10–1.14)	<0.001	1.10 (1.08–1.12)	<0.001
Number of prescriptions	1.01 (1.01–1.01)	<0.001	1.00 (1.00–1.00)	0.493

Abbreviations: CI = confidence interval, CH = Charlson, CKD = chronic kidney disease, eGFR = estimated glomerular filtration rate, and OR = Odds Ratio. ^1^ Adjusted for all variables considered in the univariate logistic regression model. ^2^ Range codified by each eGFR category: G3a between 45 and 59 mL/min/1.73 m^2^; G3b between 30 and 44 mL/min/1.73 m^2^; and G4/G5 <30 mL/min/1.73 m^2^.

**Table 3:** Contraindicated drug classes (Anatomical Therapeutic and Chemical (ATC) II level) prescriptions in patients with an eGFR <60 mL/min/1.73 m^2^.

ATC II—Drug Classes	N. of Total Prescriptions	Contraindicated Prescriptions *n* (% ^1^)
M01—Anti-inflammatory and antirheumatic products	3835	2026 (52.8)
B01—Antithrombotic agents	10,026	1917 (19.1)
A10—Drugs used in diabetes	7504	225 (3.0)
A12—Mineral supplements	800	118 (14.8)
C08—Calcium channel blockers	4441	105 (2.4)
C07—Beta blocking agents	5786	94 (1.6)
N06—Psychoanaleptics	3720	72 (1.9)
C03—Diuretics	5474	85 (1.6)
C10—Lipid modifying agents	10,133	46 (0.5)
G04—Urologicals	2880	44 (1.5)
N05—Psycholeptics	1893	33 (1.7)
J01—Antibacterials for systemic use	4932	28 (0.6)
M05—Drugs for treatment of bone diseases	1603	13 (0.8)
A02—Drugs for acid related disorders	18,405	4 (0.02)
N02—Analgesics	1581	3 (0.2)
H01—Pituitary and hypothalamic hormones and analogues	3	2 (66.7)
C09—Agents acting on the renin-angiotensin system	14,853	1 (0.01)
N04—Anti-Parkinson drugs	569	1 (0.2)
S01—Ophthalmologicals	2214	1 (0.05)

Abbreviations: ATC = Anatomic Therapeutic and Chemical, and eGFR = estimated glomerular filtration rate. Only contraindicated active substances in CKD were taken into account for each drug class (ATC II level). ^1^ Percentages calculated on the total of prescriptions for the respective drug class.

**Table 4:** Most commonly contraindicated drugs (*n* ≥ 10) in patients with CKD and the number of their prescriptions.

Drug	Patients with at Least One Contraindicated Prescription *n* = 896 (% ^1^)	Contraindicated Prescriptions *n* = 4818 (% ^2^)
Diclofenac ^3^	279 (31.1)	854 (17.7)
Acetylsalicylic acid ^3, 4^	174 (19.4)	1902 (39.5)
Etoricoxib ^3^	125 (14.0)	432 (9.0)
Aceclofenac ^3^	65 (7.3)	147 (3.1)
Celecoxib ^3^	50 (5.6)	149 (3.1)
Nimesulide ^3^	43 (4.8)	128 (2.7)
Ketoprofen ^3^	40 (4.5)	134 (2.8)
Calcium carbonate ^3^	30 (3.3)	117 (2.4)
Ibuprofen ^3^	28 (3.1)	60 (1.2)
Piroxicam ^3^	25 (2.8)	50 (1.0)
Canrenone ^3^	13 (1.5)	69 (1.4)
Ketorolac ^5^	13 (1.5)	21 (0.4)
Dexibuprofen ^3^	12 (1.3)	18 (0.4)
Metformin ^3^	10 (1.1)	164 (3.4)

Abbreviation: eGFR = estimated glomerular filtration rate. Drugs considered contraindicated according to contraindications reported in their Summary of Product Characteristics (SmPCs). For details, see Supplementary Materials, Table S1. ^1^ Percentages calculated on the total of patients with an eGFR <60 mL/min/1.73 m^2^ (*n* = 896). ^2^ Percentages calculated on the total of contraindicated prescriptions (*n* = 4818). ^3^ Patients with an eGFR <30 mL/min/1.73 m^2^ or with at least one comorbidity as reported in SmPCs. For details, see Supplementary Materials, Table S1. ^4^ Including ATC codes B01AC06 and N02BA01. ^5^ Patients with an eGFR <60 mL/min/1.73 m^2^.

## Supplementary Materials

The following are available online at https://www.mdpi.com/2077-0383/9/5/1346/s1, Table S1: List of contraindicated drugs in patients with chronic kidney disease (CKD) on the basis of the Summary of Product Characteristics (SmPC), Table S2: Characteristics of patients with chronic kidney disease (CKD) and registered creatinine values stratified by the CKD-EPI formula.
