# Supplementary material for: Chronic Kidney Disease Management in General Practice: A Focus on Inappropriate Drugs Prescriptions
Source: J Clin Med. 2020 May 4;9(5):1346. doi: 10.3390/jcm9051346 (PMC7290782; doi:10.3390/jcm9051346)
Supplement: Supplementary file 1 [file jcm-09-01346-s001.pdf]

Supplementary Materials

Table S1. List of contraindicated drugs in patients with chronic kidney disease (CKD) on the basis of the Summary of Product Characteristics (SmPC).

ATC II – Drug class	Active substance	Notes
Agents acting on the renin-angiotensin system	benazepril	eGFR <60 ml/min/1.73m^2 in combination with aliskiren
	captopril	eGFR <60 ml/min/1.73m^2 in combination with aliskiren
	cilazapril	eGFR <60 ml/min/1.73m^2 in combination with aliskiren
	enalapril	eGFR <60 ml/min/1.73m^2 in combination with aliskiren
	irbesartan	eGFR <60 ml/min/1.73m^2 in combination with aliskiren
	lisinopril	eGFR <60 ml/min/1.73m^2 in combination with aliskiren
	moexipril	eGFR <60 ml/min/1.73m^2 in combination with aliskiren
	olmesartan	eGFR <60 ml/min/1.73m^2 in combination with aliskiren
	ramipril	eGFR <60 ml/min/1.73m^2 in combination with aliskiren
	quinapril	eGFR <60 ml/min/1.73m^2 in combination with aliskiren
	telmisartan	eGFR <60 ml/min/1.73m^2 in combination with aliskiren
	trandolapril	eGFR <60 ml/min/1.73m^2 in combination with aliskiren
	zofenopril	eGFR <20 ml/min/1.73m^2
Analgesics	acetylsalicylic acid	eGFR <30 ml/min/1.73m^2 or with one of the following diagnosis (570-573, 070, 072.71, 428, 416.9) or in combination with methotrexate
	rizatriptan	eGFR <30 ml/min/1.73m^2
	zolmitriptan	eGFR <15 ml/min/1.73m^2
Antacids	aluminium compounds	eGFR <30 ml/min/1.73m^2
	calcium compounds	eGFR <30 ml/min/1.73m^2
	combinations of aluminium, calcium, magnesium compounds	eGFR <30 ml/min/1.73m^2
	magnesium compounds	eGFR <30 ml/min/1.73m^2
Antibacterials for systemic use	nitrofurantoin	eGFR <40 ml/min/1.73m^2
	sulfametoxazolo + trimetoprim	eGFR <30 ml/min/1.73m^2 or with one of the following diagnosis (570-573, 070, 072.71, 282.2)
Antigout preparations	colchicine	eGFR <30 ml/min/1.73m^2
Antihistamines for systemic use	cetirizine	eGFR <10 ml/min/1.73m^2
	levocetirizine	eGFR <10 ml/min/1.73m^2
Anti-inflammatory and antirheumatic products	aceclofenac	eGFR <30 ml/min/1.73m^2 or with one of the following diagnosis (570-573, 070, 072.71, 428, 416.9, 400-414, 440 - 440.30, 440.9, 430-434, 435.8-435.9, 436-437)
	celecoxib	eGFR < 30 ml/min/1.73m^2 or with one of the following diagnosis (570-573, 070, 072.71, 428, 416.9, 400-414, 440 - 440.30, 440.9, 430-434, 435.8-435.9, 436-437)
	dexketoprofen	eGFR <60 ml/min/1.73m^2 or with one of the following diagnosis (570-573, 070, 072.71, 428, 416.9)
	dexibuprofen	eGFR <30 ml/min/1.73m^2 or with one of the following diagnosis (570-573, 070, 072.71, 428, 416.9)
	diclofenac	eGFR <30 ml/min/1.73m^2 or with one of the following diagnosis (570-573, 070, 072.71, 428, 416.9, 400-414, 440 - 440.30, 440.9, 430-434, 435.8-435.9, 436-437);<br>in combination with misoprostol eGFR <30 ml/min/1.73m^2 and with the diagnosis of at least one of the following diseases (070, 072.71, 130.5, 573.1, 573.2, 573.3, 130.5, 570, 571.1-571.2-571.3-571.4, 571.5,428, 416.9)

	etoricoxib	eGFR <30 ml/min/1.73m^2 or with one of the following diagnosis (570-573, 070, 072.71, 428, 416.9, 400-414, 440 - 440.30, 440.9, 430-434, 435.8-435.9, 436-437)
	ibuprofen	eGFR <30 ml/min/1.73m^2 or with one of the following diagnosis (570-573, 070, 072.71, 428, 416.9)
	ketoprofen	eGFR <30 ml/min/1.73m^2 or with one of the following diagnosis (428, 416.9)
	ketorolac	eGFR <60 ml/min/1.73m^2 or with one of the following diagnosis (070, 072.71, 130.5, 573.1, 573.2, 573.3, 130.5, 570, 571.1-571.2-571.3-571.4, 571.5,428, 416.9)
	lornoxicam	eGFR <30 ml/min/1.73m^2 or with one of the following diagnosis (570-573, 070, 072.71, 428, 416.9)
	meloxicam	eGFR <30 ml/min/1.73m^2 or with one of the following diagnosis (570-573, 070, 072.71, 428, 416.9)
	naproxen	eGFR <30 ml/min/1.73m^2 or with one of the following diagnosis (428, 416.9);<br>in combination with esomeprazole eGFR <30 ml/min/1.73m^2 and with the diagnosis of at least one of the following diseases (570-573, 070, 072.71, 428, 416.9)
	niflumic acid	eGFR <30 ml/min/1.73m^2 or with one of the following diagnosis (570-573, 070, 072.71, 428, 416.9)
	nimesulide	eGFR <30 ml/min/1.73m^2 or with one of the following diagnosis (570-573, 070, 072.71, 428, 416.9)
	piroxicam	eGFR <30 ml/min/1.73m^2 or with one of the following diagnosis (570-573, 070, 072.71, 428, 416.9, 400-414, 440 - 440.30, 440.9, 430-434, 435.8-435.9, 436-437)
	tenoxicam	eGFR < 30 ml/min/1.73m^2
	tiaprofenic acid	eGFR < 30 ml/min/1.73m^2 or with one of the following diagnosis (570-573, 070, 072.71)
Antineoplastic agents	cisplatin	eGFR <60 ml/min/1.73m^2
	methotrexate	eGFR <30 ml/min/1.73m^2 or with one of the following diagnosis (570-573, 070, 072.71)
Anti-Parkinson drugs	ropinirole	eGFR <30 ml/min/1.73m^2
Antithrombotic agents	acetylsalicylic acid	eGFR <30 ml/min/1.73m^2 or with one of the following diagnosis (570-573, 070, 072.71, 428, 416.9) or in combination with methotrexate
	dabigatran	eGFR <30 ml/min/1.73m^2
	fondaparinux	eGFR <20 ml/min/1.73m^2
	nadroparin	eGFR<30 ml/min/1.73m^2 or with the diagnosis of 453.40
Beta blocking agents	nebivolol	eGFR < 30 ml/min/1.73m^2
	sotalol	eGFR < 10 ml/min/1.73m^2
Cardiac therapy	trimetazidine	eGFR <30 ml/min/1.73m^2
Calcium channel blockers	lercanidipine	eGFR < 30 ml/min/1.73m^2
	manidipine	eGFR < 10 ml/min/1.73m^2
Diuretics	canrenone	eGFR <30 ml/min/1.73m^2 (in combination with benazepril eGFR <60 ml/min/1.73m^2)
	eplerenone	eGFR <30 ml/min/1.73m^2
	hydrochlorothiazide	eGFR <30 ml/min/1.73m^2 or with one of the following diagnosis (570-573, 070, 072.71, 275.42)
	indapamide	eGFR <30 ml/min/1.73m^2
	piretanide	eGFR <60 ml/min/1.73m^2
	potassium canrenoate	eGFR <30 ml/min/1.73m^2
	spironolactone	eGFR <30 ml/min/1.73m^2
Drugs for treatment of bone diseases	alendronic acid	eGFR <30 ml/min/1.73m^2
	risedronic acid	eGFR <35 ml/min/1.73m^2
	zoledronic acid	eGFR <35 ml/min/1.73m^2
Drugs used in diabetes	acarbose	eGFR <25 ml/min/1.73m^2

	glibenclamide	eGFR <30 ml/min/1.73m^2
	glicazide	eGFR <30 ml/min/1.73m^2
	glimepiride	eGFR <30 ml/min/1.73m^2
	glipizide	eGFR <30 ml/min/1.73m^2
	metformin	eGFR <30 ml/min/1.73m^2
Immunostimulants	interferon alfa-2b	eGFR <30 ml/min/1.73m^2
Immunosuppressants	leflunomide	eGFR <60 ml/min/1.73m^2
Lipid modifying agents	bezafibrate	eGFR <60 ml/min/1.73m^2
	fenofibrate	eGFR <30 ml/min/1.73m^2
	gemfibrozil	eGFR <30 ml/min/1.73m^2
	rosuvastatin	eGFR <30 ml/min/1.73m^2
Mineral supplements	calcium compounds	eGFR <30 ml/min/1.73m^2
	magnesium compounds	eGFR <30 ml/min/1.73m^2
	potassium compounds	eGFR <30 ml/min/1.73m^2
Ophthalmologicals	brinzolamide	eGFR <30 ml/min/1.73m^2
	dorzolamide	eGFR <30 ml/min/1.73m^2
Pituitary and hypothalamic hormones and analogues	desmopressin	eGFR <30 ml/min/1.73m^2
Psychoanaleptics	duloxetine	eGFR <30 ml/min/1.73m^2
	galantamine	eGFR <9 ml/min/1.73m^2
Psycholeptics	clozapine	eGFR <30 ml/min/1.73m^2
	lithium	eGFR <60 ml/min/1.73m^2
Sex hormones and modulators of the genital system	danazol	eGFR <30 ml/min/1.73m^2
	raloxifene	eGFR <30 ml/min/1.73m^2
Urologicals	alfuzosin	eGFR <30 ml/min/1.73m^2
	vardenafil	eGFR <30 ml/min/1.73m^2

Abbreviations: ATC = Anatomic Therapeutic and Chemical, and eGFR = estimated glomerular filtration rate. All diagnoses were codified by the International Classification of Diseases code, 9th revision (ICD9).

Table S2. Characteristics of patients with chronic kidney disease (CKD) and registered creatinine values stratified by the CKD-EPI formula.

Characteristic	G3a [1] (*n* = 570)	G3b [1] (*n* = 239)	G4/G5 [1] (*n* = 87)	All patients (*n* = 896)
**Sex, *n* (%)**				
Male	200 (35.1)	76 (31.8)	39 (44.8)	315 (35.2)
Female	370 (64.9)	163 (68.2)	48 (55.2)	581 (64.8)
Median age (Q1-Q3)	75 (67-81)	81 (75-86)	83 (73-87)	77 (70-83)
**Age categories, *n* (%)**				
18-65	121 (21.2)	16 (6.7)	5 (5.7)	142 (15.8)
65-80	314 (52.3)	103 (41.8)	31 (35.6)	448 (50.0)
>80	151 (26.5)	123 (51.5)	51 (58.6)	325 (36.3)
**Comorbidities, *n* (%)**				
Atherosclerosis	113 (19.8)	56 (23.4)	19 (21.8)	188 (21.0)
Arthritis and arthrosis	320 (56.1)	136 (56.9)	47 (54.0)	503 (56.1)
Cerebrovascular disease	180 (31.6)	89 (37.2)	36 (41.4)	305 (34.0)
Dyslipidaemia	348 (61.1)	139 (58.2)	51 (58.6)	538 (60.0)
Diabetes Mellitus	195 (34.2)	89 (37.2)	38 (43.7)	322 (35.9)
Heart failure	47 (8.2)	40 (16.7)	26 (29.9)	113 (12.6)
Gout and metabolism disorders	62 (10.9)	47 (19.7)	25 (28.7)	134 (15.0)
Osteoporosis	262 (46.0)	112 (46.9)	35 (40.2)	409 (45.6)

Psychosis	253 (44.4)	114 (47.7)	40 (46.0)	389 (43.4)
Hypertension	482 (84.6)	223 (93.3)	80 (92.0)	785 (87.6)
Chronic pulmonary diseases	262 (46.0)	118 (49.4)	43 (49.4)	423 (47.2)
Malignant neoplasm	75 (13.2)	22 (9.2)	13 (14.9)	110 (12.3)
Registered CKD diagnosis, *n* (%)	103 (18.1)	97 (40.6)	65 (74.7)	265 (29.6)
Nephrologist visits [2], *n* (%)	85 (14.9)	80 (33.5)	63 (72.4)	228 (25.4)
CH index, median (Q1-Q3)	3 (1-5)	4 (2-6)	5 (3-8)	3 (1.75-6)
Number of prescriptions, median (Q1-Q3)	113 (60-187)	152 (91-229)	196 (117-65)	127 (72-205)
Number of drugs, median (Q1-Q3)	18 (12-25)	22 (15-29)	25 (20-33)	20 (13-27)

Abbreviations: CH = Charlson, CKD = chronic kidney disease, eGFR = estimated glomerular filtration rate, Q1 = first quartile, and Q3 = third quartile. [1] Range codified by eGFR category: G3a between 45 and 59 ml/min/1.73m^2; G3b between 30 and 44 ml/min/1.73m^2; and G4/G5 <30 ml/min/1.73m^2. [2] Patients with almost one nephrologist visit during the study period.
